# SANS Study of PPPO in Mixed Solvents and Impact on Polymer Nanoprecipitation

**DOI:** 10.1021/acs.macromol.1c02030

**Published:** 2022-01-18

**Authors:** Róisín A. O’Connell, William N. Sharratt, Nico J. J. Aelmans, Julia S. Higgins, João T. Cabral

**Affiliations:** † Department of Chemical Engineering, Imperial College London, South Kensington, London SW7 2AZ, United Kingdom; ‡ Buchem B.V., Minden 60, Apeldoorn 7327 AW, Netherlands

## Abstract

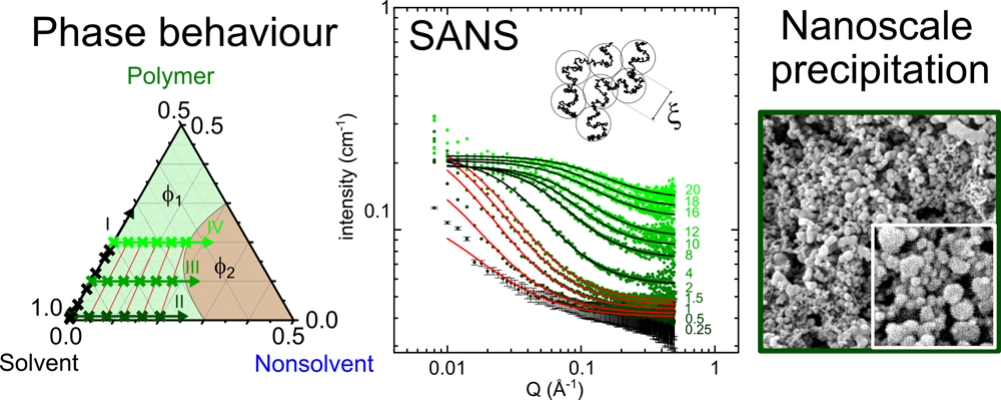

We investigate the conformation of poly(2,6-diphenyl-*p*-phenylene oxide) (PPPO) in good and mixed solvents by small-angle neutron scattering (SANS) across its ternary phase diagram. Dichloromethane was selected as a “good” solvent and heptane as a “poor” solvent whose addition eventually induces demixing and polymer precipitation. Below the overlap concentration c*, the polymer conformation is found to be well described by the polymer-excluded volume model and above by the Ornstein–Zernike expression with a correlation length ξ which depends on the concentration and solvent/nonsolvent ratio. We quantify the decrease in polymer radius of gyration *R*_*g*_, increase in ξ, and effective χ parameter approaching the phase boundary. Upon flash nanoprecipitation, the characteristic particle radius (estimated by scanning electron microscopy, SEM) is found to scale with polymer concentration as well as with nonsolvent content. Significantly, the solution volume per precipitated particle remains nearly constant at all polymer concentrations. Overall, our findings correlate ternary solution structure with the fabrication of polymer nanoparticles by nonsolvent-induced phase separation and precipitation.

## Introduction

Liquid–liquid demixing of polymer solutions is a versatile and ubiquitous manufacturing process exploited in the fabrication of polymeric membranes, scaffolds, and porous materials and particles.^1−4^ Applications of such materials range from drug delivery^5^ and food^6^ to catalysis^7^ and sensing.^8^ Demixing is generally induced by a temperature change, by the addition of a poor solvent (nonsolvent-induced phase separation, NIPS), or by a pressure change.^9^ Phase separation results in the formation and coexistence of polymer-rich and polymer-poor (solvent-rich) phases that evolve with time to eventually form the polymer matrix and “void” space, respectively.^1^

Ternary polymer/solvent/nonsolvent thermodynamics are often rationalized in terms of Flory–Huggins theory to describe NIPS,^10^ and recent simulations have coupled solvent/nonsolvent exchange and phase separation in ternary solutions^11^ and glass formation^12^ in the context of membrane formation. Demixing occurs due to an elevated free energy state of the system and proceeds via either nucleation and growth (N&G) or spinodal decomposition mechanisms, yielding characteristic cellular or bicontinuous interpenetrating structures, respectively. Considerably less is known about the conformation and dimensions of polymer chains in the one-phase region upon nonsolvent addition and the role of concentration fluctuations approaching the phase boundary or how these might be related to the resultant polymeric structures formed by demixing and precipitation.

The dimensions of a polymer chain in a binary solvent mixture can be higher or lower than those found in the pure solvents^13,14^ and depend on the interactions between the polymer and each solvent and between solvents. A mixture of two good solvents may act as a poor solvent to the polymer (cononsolvency),^15^ and conversely, two poor solvents may result in a good solvent (cosolvency),^16^ as recognized since the 1970s in terms of local solvent concentration and preferential adsorption onto the polymer.^17^ In many cases, however, ternary polymer and solvent/nonsolvent mixtures in the one-phase region can be considered in terms of a single “effective” solvent with a combined interaction parameter for the given polymer, whose variation of chain dimensions and interaction parameter in mixed solvents was first considered theoretically by Schultz and Flory.^18^

A generalized Flory–Huggins-type theory for competitive solvation has been proposed,^20^ and various polymer conformation models in mixed solvents have been reviewed recently,^21^ highlighting the scarcity of experimental data to enable their comparative examination and validation. Experimentally, small-angle neutron scattering (SANS) provides a direct measure of polymer chain dimensions, conformation, and interactions, and there are relatively few reports of polymers in mixed solvents; binary polymer solutions approaching the phase boundary as a function of temperature^22−24^ and pressure^25^ have been probed by SANS as well as mixed solvent polymer systems with light scattering;^26^ cononsolvency effects^27,28^ have been investigated and an effective χ parameter description employed.^29^ Here, we consider a ternary polymer:solvent:nonsolvent system approaching the phase boundary by addition of nonsolvent and the ensuing nanoparticle formation upon precipitation from the dilute regime.

**Figure 1:**
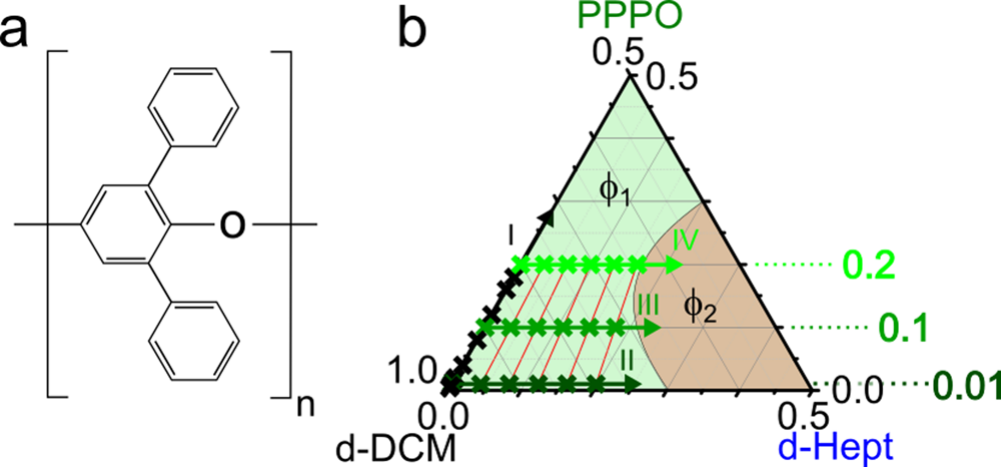
(a) Monomer structure of poly(2,6-diphenyl-*p*-phenylene oxide) (PPPO). (b) Ternary phase diagram of PPPO/dichloromethane (DCM)/heptane (exhibiting a small shift upon solvent deuteration^19^), indicating the one-phase (ϕ_1_, green) and two-phase (ϕ_2_, red) regions. Investigated compositions are shown as crosses along isopleths: (I) binary mixtures of PPPO in *d*-DCM, (II) *x*_PPPO_ = 0.01 (<c*) with increasing heptane, (III) *x*_PPPO_ = 0.1 (>c*), and (IV) *x*_PPPO_ = 0.2 with increasing heptane. Ternary mixtures (II–IV) were selected to ensure the solvent:nonsolvent ratio is consistent for each incremental addition of heptane (along red lines) regardless of PPPO concentration (*d*-DCM:*d*-Hept = 96:4, 92:8, 88:12, 84:16, and 80:20).

We previously mapped the phase behavior and demixing of poly(2,6-diphenyl-*p*-phenylene oxide) (PPPO), whose monomer structure is shown in Figure 1a, in mixed solvents dichloromethane (DCM, a good solvent) and heptane (a nonsolvent).^19^ The ternary phase diagram is depicted in Figure 1b, where the one-phase region (ϕ_1_) is shown in green and the two-phase in red (ϕ_2_). In this work, we use SANS to probe the polymer conformation and solution behavior of PPPO in deuterated solvents, *d*-DCM and *d*-heptane. We select a range of binary dilute and semidilute PPPO:*d*-DCM solutions and investigate the addition of nonsolvent *d*-heptane (at fixed polymer concentration) toward the phase boundary at selected compositions shown as crosses in Figure 1b. We seek to resolve the effect of nonsolvent addition to overall solvent quality, polymer chain dimensions, and concentration fluctuations prior to phase separation and consider the effect this may have on particle formation via precipitation.

## Experimental Section

### Materials

Poly(2,6-diphenyl-*p*-phenylene oxide) (PPPO) (*M*_n_ = 176 kg/mol, *M*_w_/*M*_n_ = 1.95, *T*_g_ = 228 °C) was used for these studies. Deuterated dichloromethane (*d*-DCM, VWR Chemicals, 99.9%, ρ = 1.36 g/cm^3^ at 20 °C^30^) and heptane (*d*-Hept, VWR Chemicals, 99.8%, ρ = 0.79 g/cm^3^, refractive index = 1.384 at 20 °C^30^) were used as solvent and nonsolvent, respectively. Ternary solutions were prepared gravimetrically according to the compositions shown in Figure 1b at room temperature (21 °C) and were stored at this temperature until use (1–2 h). Compositions were specifically selected to ensure the solvent to nonsolvent ratio is consistent for each incremental addition of heptane (along red lines) regardless of PPPO concentration.

### Small-Angle Neutron Scattering (SANS)

SANS measurements were carried out at the ISIS pulsed neutron source (Oxfordshire, UK) using the time-of-flight SANS2D diffractometer with an incident wavelength range of 2–14 Å at 10 Hz with two detectors at distances of 2.4 and 4 m from the sample, yielding an approximate wavevector range *Q* = (4π/λ)sin(θ/2) of 0.005–1 Å^–1^, where λ is the neutron wavelength and θ is the scattering angle. The samples were prepared gravimetrically and filtered (PTFE 1 μm) into quartz glass banjo cells of 1 mm path length (Hellma 120-QS) before SANS acquisition at room temperature (21 °C). MANTID software (v3.13)^31^ was used to reduce, merge, radially average, and calibrate the scattering data, which were then analyzed with SasView (v5.0.4).^32^ The coherent solvent contribution, from *d*-DCM and d*-*Hept, was subtracted from the total scattering intensity and referred to as *I*(*Q*). This background-subtracted scattering intensity contains the coherent scattering signal and the incoherent polymer background, *B*_inc_.

### Polymer Solution SANS Analysis

#### Dilute Polymer Solutions

In the dilute regime, polymer chains are isolated and unentangled. For neutral polymers under theta conditions, the contributions from attractive and repulsive excluded volume interactions are equal, resulting in ideal (unperturbed) chain dimensions (with excluded volume parameter ν = 1/2), and the overlap concentration, , corresponds to the point at which the overall concentration is equal to the pervaded concentration of the coil. While several overlap criteria have been proposed,^33,34^ we estimate the overlap concentration for this system as  2–3 w/w %, where *M*_w_ is the molecular weight (≃300 kg/mol), *N*_A_ is Avogadro’s number, and *R*_*g*_ (∼10 nm) is the expected radius of gyration of the polymer coil in the dilute limit.

SANS data along isopleth I (below c*) and isopleth II were solvent subtracted (*d*-DCM, *d*-DCM/*d*-Hept) and could be well fitted to the polymer-excluded volume model (Polymer_Excl_Vol in SasView). The corresponding form factor *P*(*Q*) was originally introduced by Benoît^35^ as
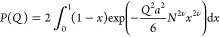
1where *a* is the statistical segment length of the polymer, *N* is the degree of polymerization, and ν is the excluded volume parameter. SasView uses a near-analytical form introduced by Hammouda,^36^ and the polymer radius of gyration
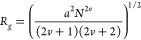
2excluded volume parameter ν, and number density of chains are readily obtained from fitting the calibrated data. Values of segment length were estimated from the measured *R*_*g*_ and molecular weight considering polydispersity and validated by comparison to previous work on poly(phenylene oxide) (PPO),^37^ as detailed in the Supporting Information.

#### Semidilute Polymer Solutions

In the semidilute regime, above c*, chains begin to interpenetrate and solution properties are influenced by the overlapping chains. These can be considered as chains of spherical “blobs”, each containing a certain number of monomers. The size of the blob is defined by the distance across which two chains are able to interact, and this length scale is called the correlation (or screening) length, ξ, which depends on solvent quality (or temperature). Along isopleth I, above c* (and below the concentrated crossover c**), and isopleth III, the solvent-subtracted data were fitted to the correlation length model (Correlation_Length model in SasView)^38^
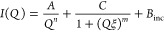
3where the first term, accounting for Porod scattering from aggregates, was not required (*A* ≡ 0) in our analysis of one-phase solutions. The second, Lorentzian, term describes the scattering from polymer chains, and ξ is the correlation length, exponent *m* describes the solvent quality (*m* = 5/3 for good and *m* = 2 for θ solvent), *C* is a scale factor, and *B*_inc_ is the incoherent scattering background from the (hydrogenous) polymer. When *m* = 2, we recover the Ornstein–Zernike expression.

#### Solutions near the Phase Boundary

Along isopleth IV and particularly at large heptane concentrations, approaching the phase boundary, an additional scattering contribution is observed and is well modeled by a second Lorentzian whose physical interpretation is discussed later. The solvent-subtracted SANS data were analyzed according to
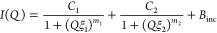
4defining an additional length scale ξ_2_ and implemented as a custom model in SasView.

#### “Effective” χ Parameter Description

For completion, we also interpret the scattering data in the framework of the random phase approximation (RPA) and Flory–Huggins thermodynamics^39−41^ customarily used to describe polymer blends in the one-phase region. The absolute coherent scattering intensity (in units of cm^–1^) of a binary mixture at equilibrium reads
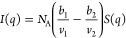
5where *b*_*i*_ are the coherent scattering lengths of the monomer units, *v*_*i*_ are their molar volumes, and *N*_A_ is Avogadro’s number. The first term defines a “contrast” factor *k* ≡ *N*_A_(*b*_1_/*v*_1_ – *b*_2_/*v*_2_) ≡ *N*_A_Δρ^2^, such that *I*(*q*) = *kS*(*q*), and *S*(*q*) is the structure factor of the mixture
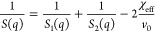
6where χ_eff_ is an effective interaction parameter (dimensionless), *S*_*i*_(*q*) is the structure factor (in units of cm^3^/mol) of each blend component *S*_*i*_(*q*) = ϕ_*i*_*v*_*i*_*N*_*i*_*P*(*q*), and *v*_0_ is a certain reference volume. Following Graessley,^42^ a number of simplifications apply for polymer solutions are made. “Component 1” is customarily the solvent (or an “effective” solvent), and the reference volume is taken as *v*_0_ ≡ *v*_*s*_, the molar volume of the solvent. The polymer volume fraction is simply ϕ_2_ ≡ ϕ, and hence, the solvent ϕ_1_ ≡ 1 – ϕ. For the solvent, *P*_1_(*q*) = 1 and *P*_2_ is the form factor of the polymer, which at intermediate *q* values can be written as . Using a Zimm representation (1/*I* vs *q*^2^), the χ_eff_ parameter can be readily estimated from the low *q* intercept as
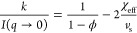
7For ternary mixtures, such as a polymer in mixed solvents, the treatment is evidently more complex and one can tentatively define an “effective solvent” medium whose scattering length density *b*_1_ and molar volume *v*_1_ ≡ *v*_*s*_ are computed as the weighted averages of the individual solvent components. An effective χ_eff_ can thus be evaluated as a function of polymer concentration and solvent/nonsolvent ratio.

### Flash Nanoprecipitation (FNP)

#### Particle Preparation

PPPO nanoparticles were generated by flash nanoprecipitation (FNP),^4,43,44^ which rapidly impinges opposing jets of dilute polymer solution and nonsolvent within a confined geometry, typically a confined impinging jet (CIJ) mixer or a multi-inlet vortex mixer (MIVM). Typical volumetric flow rates of ∼1 mL/s per inlet fluid, in typical operation of a CIJ mixer,^45^ yield jet velocities of ∼1 m/s, which result in the rapid mixing (∼milliseconds), quenching the mixture in the two-phase region, and cause a chain-to-globule transition of dilute chains. Homogeneous nucleation and diffusion-limited growth/aggregation of globules ensues, leading to the formation of a nanoparticle suspension. The selected inlet polymer solution compositions correspond to those along isopleths I (below c*) and II, ensuring that both the polymer solution (PPPO:DCM) and the nonsolvent (heptane) have similar viscosities and thus momenta of impinging jets to ensure rapid energy dissipation and uniform mixing. Further details are provided in Supporting Information Figure S1.

#### Scanning Electron Microscopy (SEM)

Dry polymer nanoparticles were mounted on carbon tape, coated with ∼10 nm of gold, and imaged at 10 kV with a typical working distance of 4–5 mm using a Zeiss Auriga Crossbeam scanning electron microscope (SEM).

## Results and Discussion

### Polymer in Good Solvent: Binary PPPO:*d*-DCM Mixtures

SANS experiments were first carried out on binary solutions of PPPO in *d*-DCM over a range of concentrations 0.25–20 w/w % PPPO with the aim to cover the dilute, semidilute, to concentrated regions.

**Figure 2:**
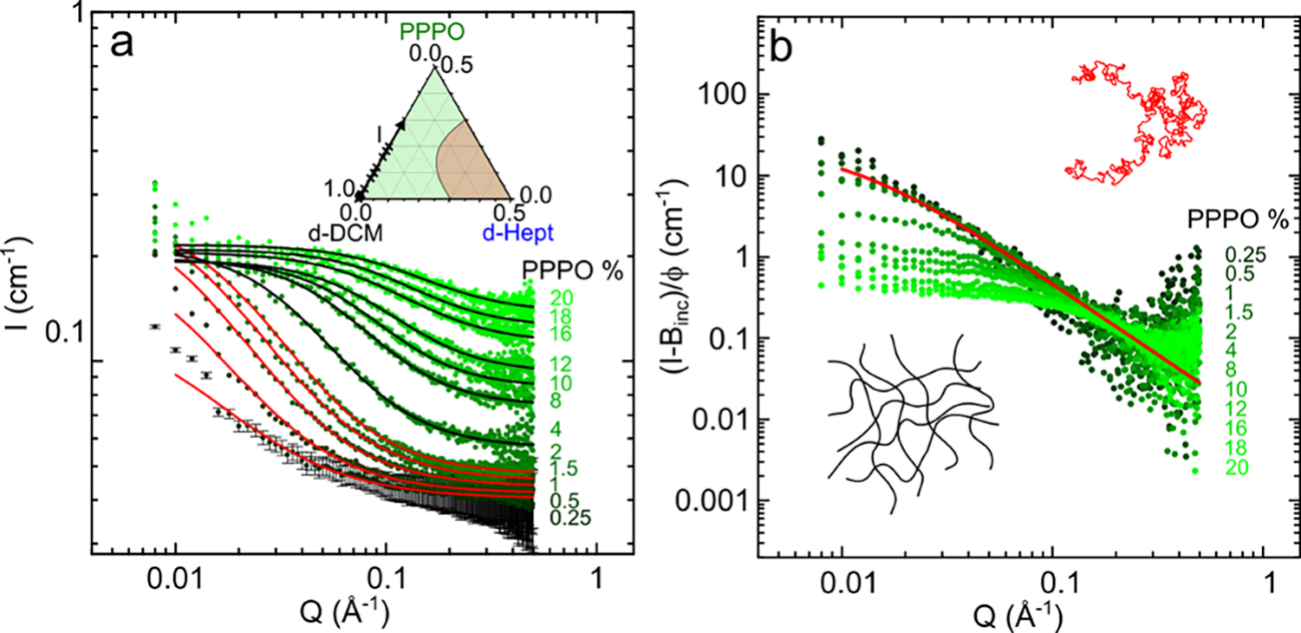
(a) Solvent-subtracted SANS intensity for binary mixtures of 0.25–20 w/w % PPPO in *d*-DCM (isopleth I) with solid lines indicating model fits. Red lines indicate solutions below c* (≤2%) fitted by the polymer-excluded volume model and black lines using the correlation length model. Error bars are included for the lowest PPPO concentration. (Inset) Ternary phase diagram and associated isopleth (I) for this data set. (b) SANS scattering intensity after subtraction of both the solvent background and the polymer incoherent contribution (*B*_inc_) for binary mixtures of PPPO in *d*-DCM (isopleth I) and then divided by PPPO volume fraction. Compositions below c* collapse onto one curve (red line shown for *R*_*g*_ ≃ 90 Å and exponent −5/3). (Insets) Dilute polymer chain (red, ≤c*) and polymer network (black, ≥c*).

Figure 2a shows the solvent-subtracted SANS data from the binary PPPO:*d*-DCM solutions (along isopleth I, inset) with polymer w/w % labeled to the right of each data set. Background contributions from the incoherent scattering from the polymer and coherent scattering from the solvent are detailed in Supporting Information Figures S2 and S3. The scattering length densities for PPPO and *d*-DCM were calculated to be 2.27 × 10^–6^ and 3.73 × 10^–6^ Å^–2^ respectively, and therefore, the contrast factor (Δρ^2^) was found to be 2.14 × 10^–12^ Å^–4^, as detailed in the Supporting Information.

The statistical errors decrease with increasing polymer concentration, and error bars have been included for the lowest concentration data (0.25 w/w % PPPO) to illustrate the largest possible errors. The incoherent background increases linearly with polymer concentration with a gradient of 0.48(5) cm^–1^ for neat PPPO (in line with ∼0.5 cm^–1^ for many polymers^46^); the intercept at 0.037(7) cm^–1^ corresponds to the coherent scattering of the *d*-DCM (see Supporting Information Figure S3). Data fits are shown as solid lines in Figure 2a: red lines indicate solutions in the dilute regime, analyzed by the polymer-excluded volume model (eqs 1 and 2), and black lines show data in the semidilute/concentrated regimes, analyzed using the correlation length model (eq 3). Some low *Q* deviations are likely caused by background and forward scattering contributions, and the high *Q* uncertainties are associated with lower statistics characteristic of polychromatic SANS at the tails of the neutron velocity distributions.

Figure 2b shows the coherent-only SANS data along isopleth I following background subtraction divided by the volume fraction contribution of the polymer in solution. Below c*, the scattering data are expected to collapse onto a single curve, which holds up to 2 w/w % PPPO, as shown by the single solid red line. The red schematic illustrates an isolated Gaussian coil in solution. Above c* (depicted by a black polymer mesh), as the polymer chains begin to overlap with one another, the polymer density per unit volume increases and the scattering intensity no longer collapses; in addition, interactions between adjacent chains contribute to the scattering signal.

**Figure 3:**
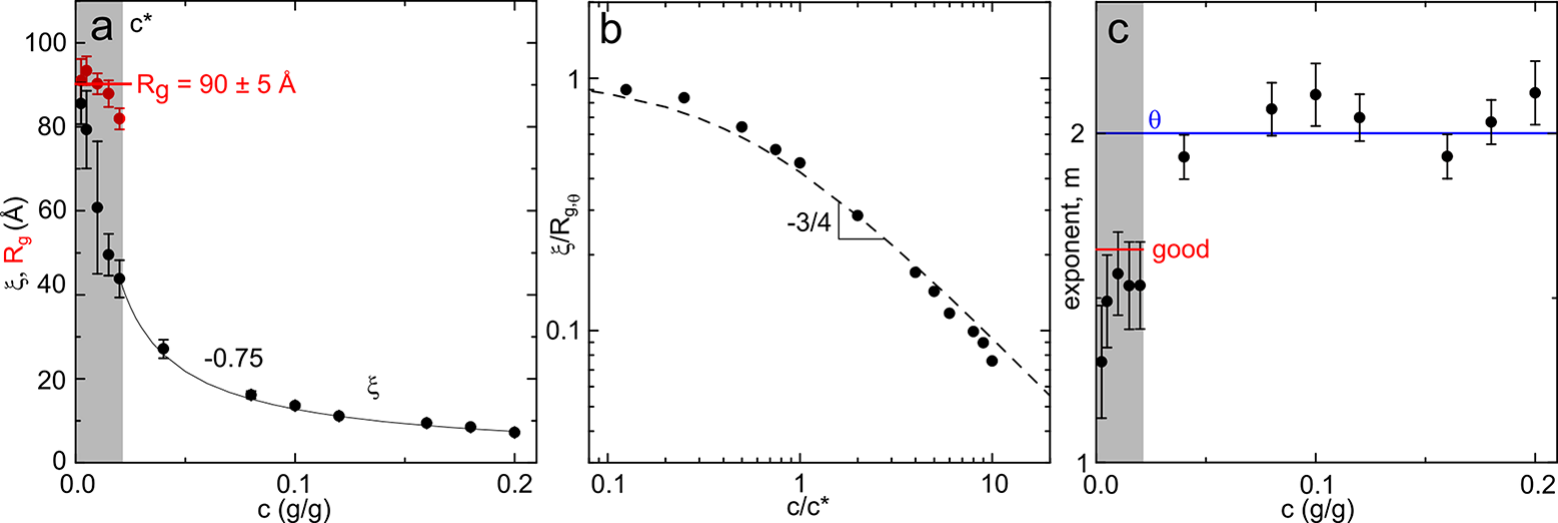
Solution parameters extracted from PPPO:*d*-DCM scattering data shown in Figure 2a along isopleth I. (a) Radius of gyration (*R*_*g*_, red) below c*, computed using the polymer-excluded volume model, and correlation length (ξ, black), from the correlation length model, with respect to PPPO concentration. Shaded area indicates the dilute region, *c* ≲ c*. (b) Reduced correlation length (ξ/*R*_*g*_) data from isopleth I with respect to reduced polymer concentration (*c*/c*). Dashed black line is a fit to (1 + *βc*/c*)^α^, where β = 2 and α = −3/4. (c) Change in Lorentzian exponent (*m*), characterizing the polymer–solvent interaction, with respect to polymer concentration. Shaded area indicates dilute regime with associated change from good (*c* ≤ c*) to approximately θ (*c* > c*) solvent.

Figure 3 summarizes the fitted parameters obtained from the black and red solid lines in Figure 2a for binary mixtures of PPPO:*d*-DCM at the concentration range investigated. Figure 3a shows the chain dimensions as a function of PPPO concentration, where the red circles show the *R*_*g*_ estimated for dilute solutions (and excluded volume model) and black circles indicate ξ, estimated from the correlation length model (for completion, fits to the correlation length model for binary solution data below c* are shown in Supporting InformationFigure S4a). The *R*_*g*_ of PPPO in good solvent *d*-DCM is found to be 90 ± 5 Å. Approaching the cross-over c* ≃ 2 w/w % PPPO, the *R*_*g*_ decreases slightly, indicating the onset of chain contraction in the semidilute regime.

As expected, above c*, the correlation length ξ decreases with polymer concentration, as the interchain distance decreases. The observed scaling law of ξ ≈ *c*^–3/4^ agrees with the expected scaling for semidilute polymer solutions in a good solvent,^47,48^ predicting a concentration dependence of ξ ∝ *c*^–ν/(3ν–1)^, so that ξ ∝ *c*^–3/4^ when ν = 0.6, the expected value for semidilute polymer solutions in a good solvent. Figure 3b shows the reduced correlation length, ξ/*R*_*g*_, of the data from Figure 2a for binary mixtures of PPPO:*d*-DCM (isopleth I) in the dilute and semidilute regimes as a function of the reduced polymer concentration *c*/c*. A line of best fit (dashed black line) is shown of the form (1 + *βc*/c*)^*α*^,^49^ where β = 2 and α = −3/4.

Figure 3c shows the change in the exponent for the fitted data in Figure 2a with respect to the PPPO solution concentration. This exponent value is characteristic of polymer–solvent interactions, and *m* (≡ 1/ν) = 5/3 (red horizontal line) is indicative of the good solvent regime. From the dilute to semidilute regimes, *m* approaches 2 (blue horizontal line), as expected toward concentrated solutions.

### Ternary Solutions of PPPO:*d*-DCM:*d*-Heptane

We next consider SANS measurements for ternary solutions of PPPO in good solvent *d*-DCM with added poor solvent *d*-heptane. PPPO concentrations (1, 10, and 20 w/w %) below and above c* were selected, and *n*-heptane was added incrementally to approach the phase boundary (isopleths II, III, and IV). The experiments were carried at *fixed* polymer content, effectively “exchanging” the good for poor solvent.

**Figure 4:**
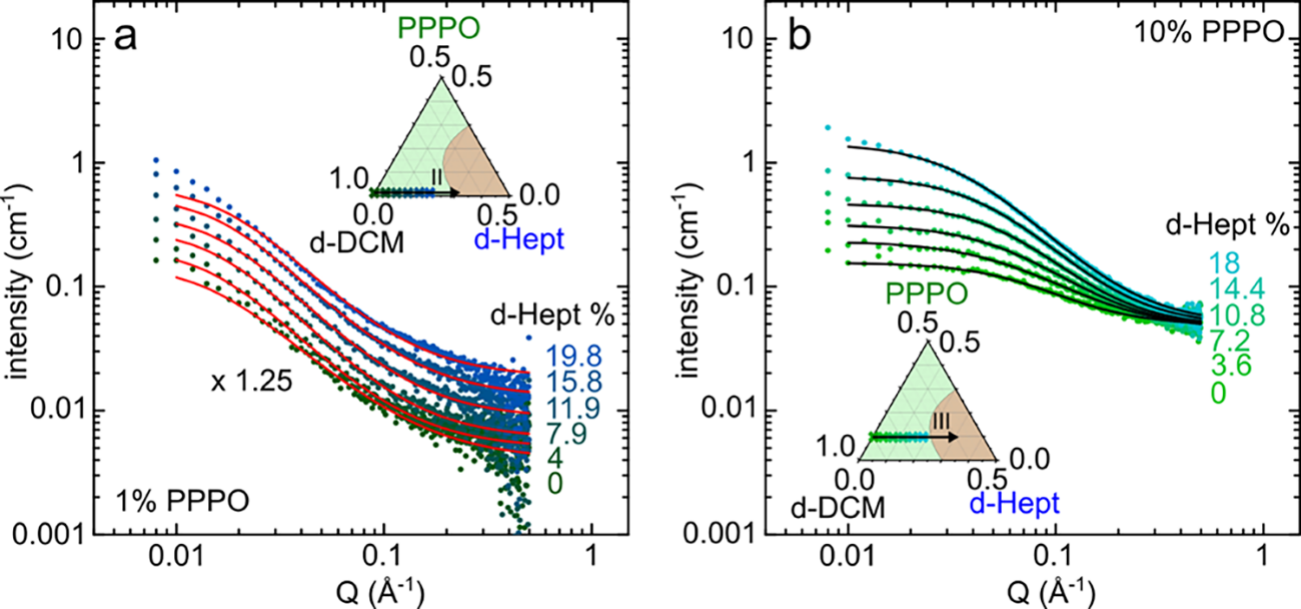
(a) Solvent-subtracted SANS intensity for ternary mixtures of 1 w/w % PPPO:*d*-DCM:*d*-Hept (isopleth II) as a function of added *d*-heptane (indicated on the right) scaled by a factor of 1.25× from the 0% *d*-heptane profile. Red lines are fits to the polymer-excluded volume model (dilute regime). (Inset) Associated isopleth (II) for these compositions. (b) Solvent-subtracted SANS intensity for ternary mixtures of 10 w/w % PPPO:*d*-DCM:*d*-Hept (isopleth III); solid black lines are fits to the correlation length model (semidilute regime).

Figure 4a shows the solvent-subtracted scattering intensity of 1 w/w % PPPO:*d*-DCM:*d*-heptane solutions (dilute regime, along isopleth II, inset) with heptane w/w % indicated on the graph. For clarity, each curve was scaled by a factor of 1.25 from the 0 w/w % heptane data with respect to increasing heptane concentration. Figure 4b shows the data for 10 w/w % PPPO:*d*-DCM:*d*-heptane solutions (in the semidilute regime, along isopleth III, inset). The coherent scatterings of *d*-DCM (nearly constant at ≃0.04 cm^–1^) and *d*-heptane (≃ 0.1 cm^–1^ with non-negligible *Q* dependence) were subtracted for the total scattering intensity in the appropriate ratios. Scattering length densities (5.49 × 10^–6^ Å^–2^ for *d*-heptane) and relevant backgrounds (Figure S2) are detailed in the Supporting Information. Both data sets in Figure 4 are expected to converge at high *Q*, corresponding to the incoherent background of the 1 or 10 w/w % PPPO, although the former do not reach this background level within the measured *Q* range (for clarity, unscaled data for 1 w/w % are shown in Figure S4b and S4c). In both cases, the overall scattering intensity increases with increasing heptane concentration within this *Q* range, associated with chain contraction in the dilute regime (effectively “shifting” the profile to higher *Q*), and with the increase in correlation length ξ in the semidilute regime, above c*. The data are well described by, respectively, the polymer-excluded volume model (red lines) and the correlation length model (black lines).

**Figure 5:**
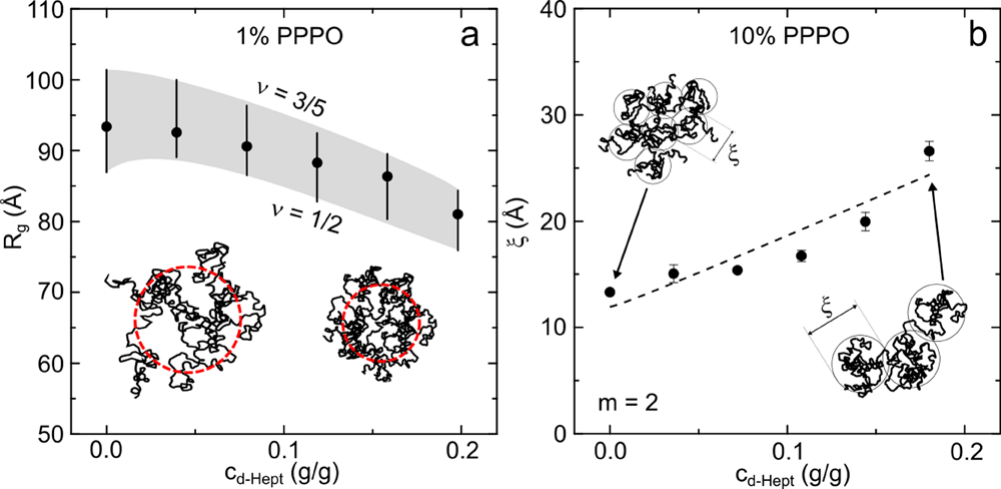
Solution parameters extracted from the data shown in Figure 4: (a) decrease in *R*_*g*_ in 1 w/w % ternary mixtures PPPO:*d*-DCM:*d*-Hept (isopleth II) upon *d*-heptane addition. Data points show best *R*_*g*_ fits (with free exponent ν), and shaded area delineates the variation of *R*_*g*_ when exponent *m* is fixed from good (*m* = 5/3) to θ (*m* = 2) solvent. Illustrations show as-expanded coil in good solvent and chain contraction by nonsolvent. (b) Change in ξ for ternary mixtures of 10 w/w % PPPO:*d*-DCM:*d*-Hept (isopleth III) with increasing *d*-heptane concentration, well described by an exponent *m* = 2. Schematics show correlation “blobs” whose size increases upon heptane addition.

Figure 5 summarizes the corresponding fitting parameters. Figure 5a shows the decrease in the *R*_*g*_ in the dilute (1 w/w %) PPPO solution, along isopleth II, of up to ≃15%, associated with chain contraction induced by the nonsolvent. The scaling exponent, ν = 3/5 for good solvent, is expected to decrease toward ν = 1/2 and possibly beyond. However, the value and uncertainty of ν is sensitive to the subtraction of the coherent scattering contribution from the solvents and the incoherent contribution from the polymer (and possible volume of mixing changes). While our data are compatible with this ν decrease, we verify the robustness of the *R*_*g*_ decrease as follows. The polymer-excluded volume model, eqs 1 and 2, was used to obtain *R*_*g*_, keeping ν as a free parameter, corresponding to the black data points; then, ν was constrained at 3/5 or 1/2 for further fits to the data and the resulting *R*_*g*_ estimated, as shown by the shaded gray band. The *R*_*g*_ reduction is thus quantified, despite some uncertainty in ν, as the solvent quality decreases. Estimating the polymerization index, *N*, and segment length *a*, we approximate *R*_*gθ*_ ≃ 60 Å (detailed in Supporting Information), indicating that the chain, although partially collapsed, remains in a “good” solvent range, consistent with the distance to the stability line shown in Figure 1b.

Figure 5b shows the change in ξ with respect to heptane concentration for solutions with 10 w/w % PPPO, along isopleth II, computed from the correlation length model, eq 3. This semidilute solution is well above c*, and the estimated correlation length, ξ, is associated with the network of overlapping polymer chains, specifically the size of a blob, in the context of the de Gennes’ blob model, representing the average distance between two chains.^47,50,51^ In a good solvent, polymer chains are swollen (ν = 3/5) inside the blobs, while for larger dimensions, the blobs become the elementary units and scaling for concentrated solutions holds (ν = 1/2). We find that ξ increases almost linearly as a function of heptane concentration, almost doubling in size. The fitting exponent was not fixed for this analysis, and *m* = 2 in eq 3 was consistently found to yield the best description of the data. We interpret this increase in ξ as due to the decrease in solvent quality and associated local conformational change that causes the distance between chains to effectively increase.

**Figure 6:**
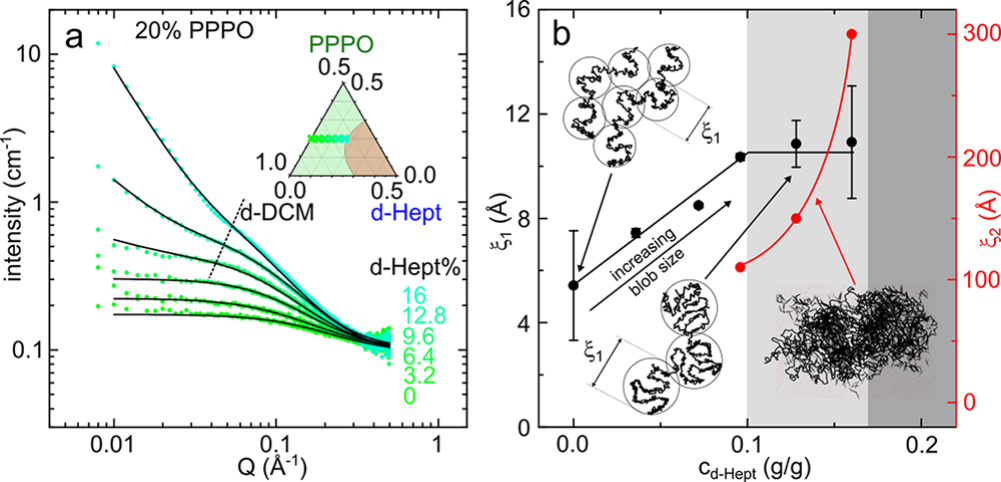
(a) SANS intensity for 20 w/w % PPPO:*d*-DCM:*d*-Hept solutions (isopleth IV) after subtraction of the solvent contribution for added *d*-heptane 0–16 w/w % indicated. Solid black lines are fits to the correlation length model, eq 3. Highest three heptane concentrations (9.6, 12.8, and 16 w/w %) were fitted using an extension of the correlation length model, eq 4, to account for the low-*Q* upturn (before dashed line). (Inset) Ternary phase diagram and associated isopleth (IV). (b) Change in correlation length ξ_1_ (black) for data shown in a with heptane concentration; in addition, values for the additional length scale of the second Lorentzian, ξ_2_ (red) are shown with a guide to the eye. Shaded areas correspond to the onset (light gray) and demixing (dark gray) boundaries.

Figure 6a shows the solvent-subtracted SANS data from the ternary 20 w/w % PPPO:*d*-DCM:*d*-heptane solutions (along isopleth IV, inset) with added *d*-heptane from 0 to 16 w/w %. As expected, the spectra converge at high *Q*, corresponding to the incoherent background intensity for 20 w/w % PPPO. As above, the scattering intensity increases with *d*-heptane addition, approaching the phase boundary; the solutions in this region are very viscous, nearly gel-like. Initial nonsolvent addition results in an increased blob size, well described by the correlation length model (eq 3) and plotted in Figure 6b. At higher concentration, viz. 9.6–16 w/w % *d*-heptane, an additional lower *Q* contribution emerges, as indicated by the dashed line in Figure 6a, and the data were instead described by a double correlation length model (eq 4). The origin of this additional contribution is not entirely clear to us, and the scattering profile is reminiscent of certain cross-linked gel systems. Temporally slicing the SANS spectra indicates that the signal is not evolving with time, as would be expected for the earlier stages of the demixing and coarsening process (Supporting Information Figure S5); yet, for the highest two concentrations of *d*-heptane, some incipient optical turbidity can be observed. We thus rationalize this contribution in terms of the polydispersity of the polymer, which results in cloud and shadow curves and thus more complex demixing stages, or possibly due to the incipient precipitation of the larger *M*_w_ tail of the distribution, effectively fractionating the solution by dropwise nonsolvent addition. We cannot, however, rule out a more complex behavior related to concentration fluctuations in this ternary mixed-solvent system.

Figure 6b depicts the correlation length ξ_1_ (black) and the descriptive parameter ξ_2_ (red). As noted by Graessley,^42^ these small sizes obtained with the Ornstein–Zernike expression should not be interpreted as a concrete physical length in the solution. With this caveat, we note that ξ_1_ increases with the addition of heptane up to 9.6 w/w %, at which point it increases more gradually or plateaus within measurement uncertainty. The red solid circles show a divergent relationship between ξ_2_ and *d*-heptane concentration approaching the two-phase region, and the dark shaded area corresponds to the location of the phase boundaries, while the lighter shaded area indicates the presence of the two scattering contributions. Overall, the results agree well with the macroscopic phase diagram.

#### “Effective” χ_eff_ Estimates

**Figure 7:**
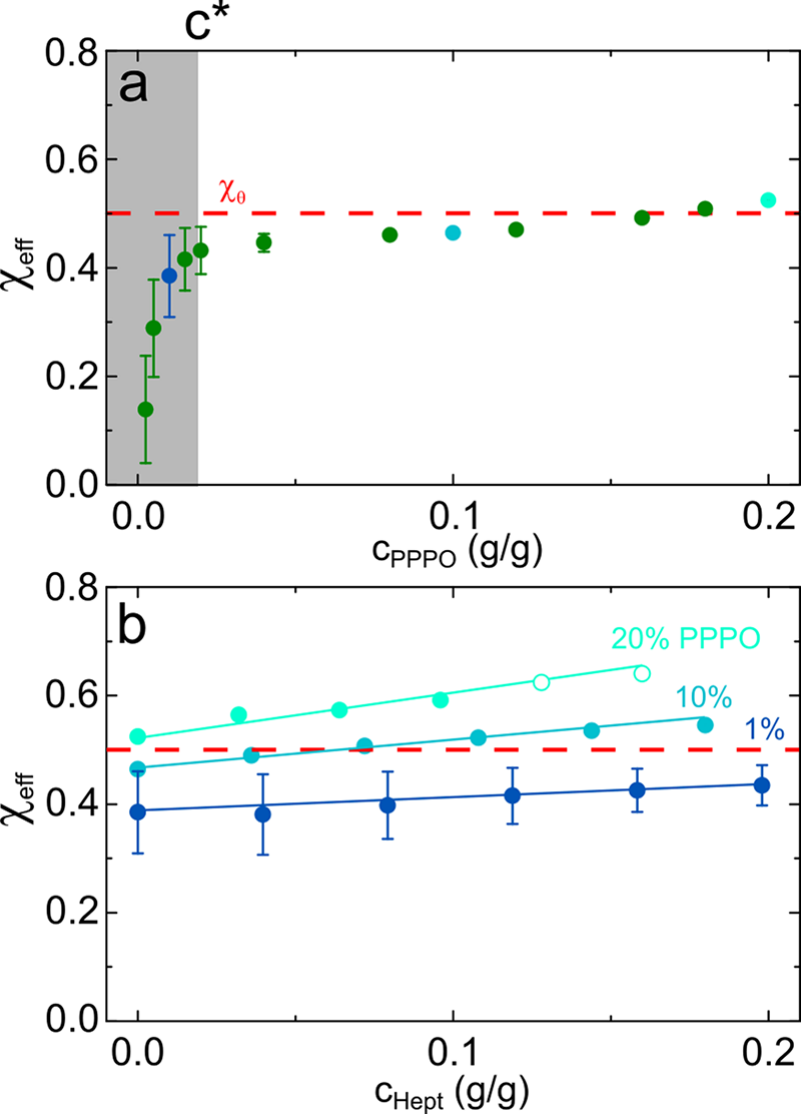
χ_eff_ values estimated for (a) binary solutions of PPPO:*d*-DCM from fits to SANS data in Figure 2 (concentrations below c* are in the shaded band) and (b) ternary solutions of PPPO:*d*-DCM:*d*-heptane from data in Figures 4 and 6. Theta condition (χ_θ_ = 0.5) is show by red dashed lines.

We next employ eq 7 as a mean to obtain a χ_eff_ parameter from the low *q* intercept of a Zimm representation (1/*I* vs *q*^2^) of the coherent scattering data (following solvent and polymer background subtraction). The “effective solvent” medium is defined to have a scattering length density *b*_1_ and molar volume *v*_1_ ≡ *v*_*s*_ computed as the weighted average (by volume fraction) of the individual solvent components, as detailed in Supporting Information Figure S6. Figure 7a plots χ_eff_ as a function of either PPPO or added nonsolvent concentration (at three fixed PPPO concentrations). An alternative mean-field treatment for semidilute polymer solutions^52^ (previously employed to characterize aqueous PNIPAM solutions^29,53^) computes χ_eff_ from the correlation length ξ and yields similar results. For the 20% PPPO solutions, we take ξ_1_ as the characteristic correlation length of the solution (since the additional lower *q* component is associated with demixing).

Low PPPO concentrations in DCM exhibit χ_eff_ < 0.5, characteristic of a polymer in good solvent, and χ_eff_ approaches 0.5 above c*, indicative of theta conditions.^54^ At all fixed PPPO concentrations, addition of heptane increases χ_eff_; for 1% PPPO, below c*, the effect is relatively small (within measurement uncertainty) and χ_eff_ does not reach 0.5. We note however that this treatment shares the limitations of the underpinning Flory–Huggins and RPA assumptions, which may not rigorously hold for very dilute solutions.^42^ Further, in mixed solvents, the lower number density of chains and proportionally large amount of good solvent present may lead to spatial heterogeneity in the concentration field.^20^

Above c*, we find that χ_eff_ increases above 0.5 upon nonsolvent addition, which occurs at a lower heptane fraction for the higher PPPO concentration or proportionally a lower fraction of good solvent present. However, in a ternary component system at a fixed temperature, the critical point exists at a specific polymer concentration, and therefore, values of χ_eff_ in Figure 7b exceeding 0.5 do not necessarily indicate demixing but rather a progressively lower solvent quality. The low *Q* upturn in the scattering profile shown in Figure 6a and the onset of optical cloudiness do indicate a small shift^19^ of the phase boundaries toward lower heptane concentration, likely due to deuteration effects (and the smaller length scales probed by SANS).

### Structure and Morphology of Precipitated Nanoparticles

**Figure 8:**
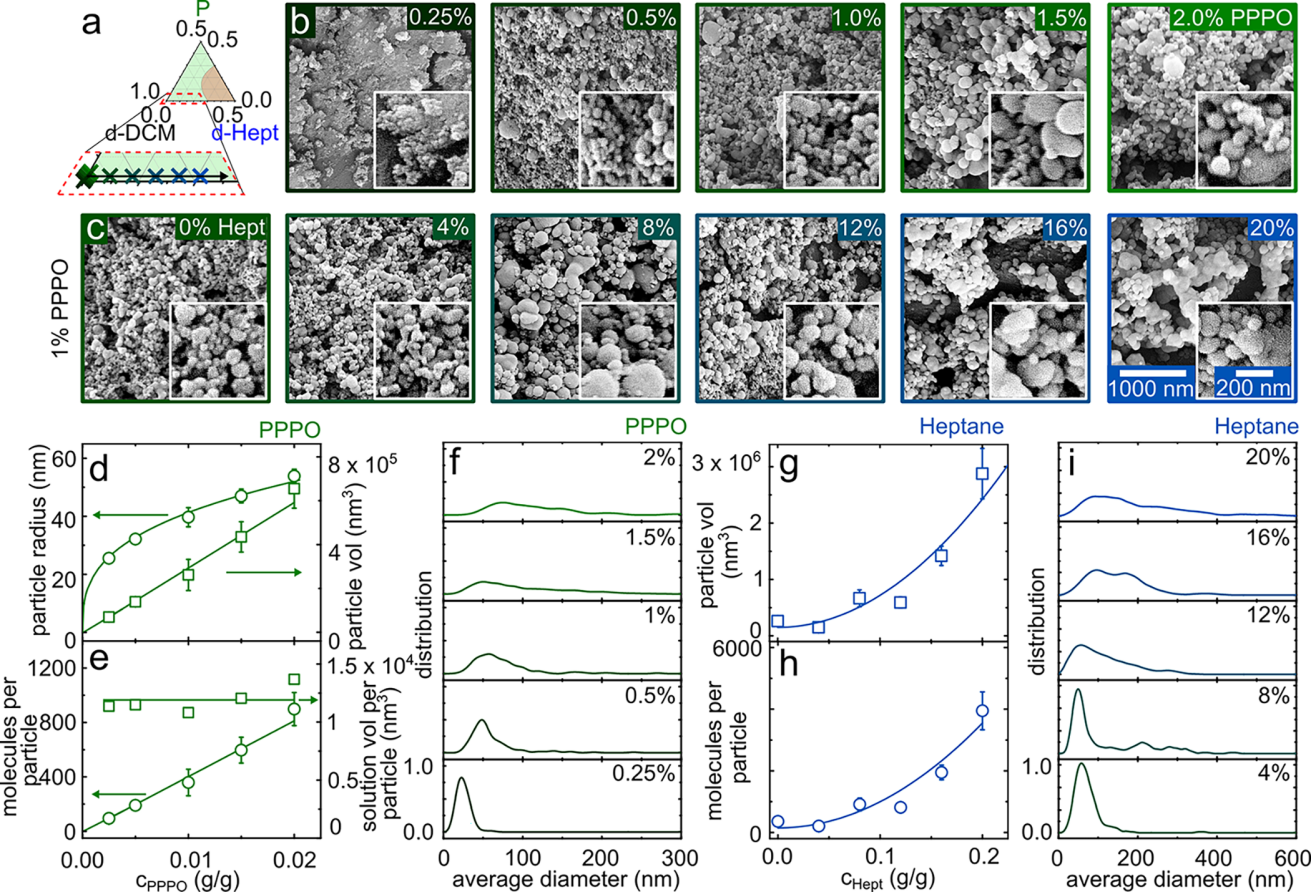
(a) Solution compositions employed in FNP: binary mixtures of PPPO:DCM below c* and ternary mixtures of PPPO:DCM:Hept (isopleth II) at 1 w/w % PPPO. (b) SEM images of PPPO nanoparticles (NP) obtained from PPPO:DCM solutions with increasing PPPO concentration (indicated on the top right of each image). (c) SEM images of NP obtained from 1 w/w % PPPO:DCM:heptane solutions with increasing heptane concentration (indicated). (d) Average particle radius (open circles) and volume (open squares); error bars are standard deviation computed from 150 particles. (e) Estimated average number of PPPO molecules per NP (open circles) and solution volume corresponding to one particle (open squares) for NP shown in b. (f) NP size distribution as a function of PPPO concentration. (g) Average particle volume corresponding to the images in c with increasing heptane concentration. (h) Estimated average number of PPPO molecules per NP. (i) NP size distribution as a function of heptane concentration.

In order to relate the solution structure to particle formation, homogeneous PPPO solutions below c* were then plunged into the two-phase region and precipitated via flash nanoprecipitation (FNP), as described in Supporting Information Figure S1. Upon chain collapse, NPs of prescribed dimensions form by aggregation before a kinetic arrest is reached within the nonsolvent (heptane) reservoir. Figure 8a displays the selected PPPO solution composition inputs using binary mixtures (isopleth I) below c* and ternary mixtures for 1 w/w % PPPO (isopleth II). Figure 8b and 8c shows SEM images of the corresponding granular PPPO NPs, and the inset shows an enlarged image of each panel. Initial visual inspection indicates that the particle size increases with increasing polymer and heptane concentration. Figure 8d shows the computed average NP radius *R*_NP_ and volume *V*_NP_ corresponding to the images in Figure 8b as a function of polymer concentration of the input solution. The resulting *R*_NP_ ≃30–60 nm and are thus ∼3–6 times larger than the polymer *R*_*g*_. The particle volume *V*_NP_ (open squares) is found to increase linearly with polymer concentration, as the radius *R*_NP_ increases with a power law of ∼1/3.

Figure 8e shows the calculated number of polymer molecules per particle *N*_NP_ (open circles), estimated from the ratio of *V*_NP_ and PPPO molecular volume (calculated as *V* ≃ *M*_w_/(*ρN*_A_)). A linear relation is also found with the initial PPPO concentration, and each NP comprises approximately 100–1000 molecules. We then estimate the initial solution volume that corresponds to the formation of a single polymer nanoparticle. Given the linearity of *V*_NP_, we expect to find a correlation between the number density of polymer molecules (*N*_d_ ≡ *N*/*V*) in solution, which evidently scales with polymer concentration, and the resulting nanoparticle volume *V*_NP_. Given that *N*_NP_ and *V*_NP_ have been measured and the polymer concentration is known, the corresponding solution volume per particle can be readily calculated. Within experimental uncertainty, we find that each nanoparticle, regardless of the initial polymer concentration, arises from the *same* solution volume of ≃1.2 × 10^4^ nm^3^.

Evidently, NP size increases while *R*_*g*_ decreases (within the dilute regime) and ξ decreases beyond c* with polymer concentration, and thus, a simple correlation between these length scales is not expected. NP dimensions appear primarily dependent on the initial solution concentration, which is consistent with the fact that FNP operates in the dilute regime, and thus, the nanoparticle size should scale with the number density of chains (which then rapidly collapse and aggregate). Assuming a small variation in solution viscosity change within the dilute concentration range, the time scales for aggregation should be comparable, and this results in a constant liquid volume forming each individual NP regardless of the initial polymer concentration. Increasing the polymer concentration therefore increases the *N*_d_ of chains and thus *V*_NP_. The constant solution volume associated per NP appears to be consistent with this physical picture and compelling. This volume is, however, expected to depend on the polymer/solvent/nonsolvent interactions, chain size, and FNP flow rate and geometry and is thus likely system dependent (although this number provides an interesting benchmark, which holds approximately for other systems below c*, e.g., polystyrene/tetrahydrofuran/water^55^).

Figure 8f shows the size distribution of the nanoparticles as PPPO concentration increases. The peak broadens with increasing concentration, as NP polydispersity increases, which could be associated with the small viscosity dependence adversely affecting the mixing efficacy and aggregation; further, proximity to c* may favor network formation over single-chain collapse, in turn promoting aggregation.^56^Figure 8g and 8h shows the average particle volume *V*_NP_ and the estimated PPPO molecules, respectively, per particle with respect to the initial heptane concentration. Both appear proportional to , which is likely associated with the proximity to the phase boundary of the input stream, leading to a greater demixing driving force and/or fast aggregation. This result contrasts with some FNP observations for which the proximity to the phase boundary was found to result in smaller particle sizes and associated with earlier kinetic arrest.^43^ However, these employed water as nonsolvent, which is thought to impart charge stabilization to NPs due to its high dielectric constant, whereas precipitation by low dielectric nonsolvents (e.g., organic) is more likely to result in aggregates.^4,57^ This appears to be the case for higher initial heptane concentrations especially, for which the NP size distribution becomes particularly broad (Figure 8i).

## Conclusions

The solution structure of poly(2,6-diphenyl-*p*-phenylene oxide) was investigated in mixed (good and bad) solvents by SANS in the one-phase region of its ternary phase diagram and related to the nanoparticles formed upon flash nanoprecipitation (FNP) in the poor solvent. Deuterated DCM and heptane were selected as the good/poor or solvent/nonsolvent pair for SANS, and their hydrogenous counterparts were employed in FNP experiments. The overlap concentration (c*) of PPPO in DCM was estimated to be 2 w/w %, and scattering data for concentrations below this c* were found to be well described by the polymer-excluded volume model, with excluded volume parameter ν = 3/5, indicative of a good solvent, and a radius of gyration (*R*_*g*_) of approximately 90 Å. For binary mixtures above c*, a Lorentzian profile with an excluded volume parameter ν = 1/2 (theta solvent) fit the scattering data best, and the estimated correlation length decreased with polymer concentration, as expected from scaling theory.

Ternary mixtures in the dilute regime were also fitted with the polymer-excluded volume model, but a decrease in *R*_*g*_ of ∼15% was observed as the solvent quality decreases near the phase boundary. Conversely, an increase in the screening length ξ was found in the semi-dilute regime with the addition of nonsolvent and attributed to local chain collapse and therefore an increase in concentration blob sizes in solution. In the concentrated regime, a second Lorentzian model was used to describe the low *Q* upturn, seen as the heptane concentration increases, and the evolution of the characteristic dimensions was related to the proximity to the phase boundary.

Polymer nanoparticles were successfully formed by FNP from dilute solutions previously investigated by SANS. The NP radius was found to scale with , meaning that the nanoparticle volumes scale linearly with concentration, *V*_NP_ ≈ *c*_PPPO_, in this regime. Estimating the number density of chains in the feed solution, our data indicate that each NP arises from the same initial solution volume of 2.5 × 10^7^ nm^3^, regardless of polymer concentration. However, we find that NP polydispersity does increase as both the polymer concentration and the heptane doping increase in the original feed solution, likely caused by increased aggregation.

## Supporting Information Available

Schematic and parameters for NP fabrication by flash nanoprecipitation (FNP); SANS spectra for solvents and mixed solvents; polymer solution SANS backgrounds and estimate of the incoherent scattering contribution of PPPO; time-resolved SANS data for a representative mixture near the stability line; estimates of chain dimensions, scattering length densities, and SANS contrast prefactor (PDF)
