# Supplementary material for: SANS Study of PPPO in Mixed Solvents and Impact on Polymer Nanoprecipitation
Source: Macromolecules. 2022 Jan 18;55(3):1050–9. doi: 10.1021/acs.macromol.1c02030 (PMC9007525; doi:10.1021/acs.macromol.1c02030)
Supplement: Supplementary file 1 — ma1c02030_si_001.pdf [file ma1c02030_si_001.pdf]

Supplementary Information

SANS study of PPPO in mixed solvents and impact on polymer nanoprecipitation

Róisín A. O'Connell[a], William N. Sharratt[a], Nico J. J. Aelmans[b], Julia S. Higgins[a], and João T. Cabral[a,*]

[a] Department of Chemical Engineering, Imperial College London, London, SW7 2AZ, U.K.
[b] Buchem B.V. Minden 60, 7327 AW Apeldoorn, Netherlands
[*]j.cabral@imperial.ac.uk

1 Flash nano-precipitation

Flash nano-precipitation (FNP, figure S1) is a technique capable to produce nanoparticles (NP) by the rapid displacement of a solvent with a nonsolvent by rapid mixing within confinement.[1,2] In the present work, 1 mL PPPO solutions, binary (0.25, 0.5, 1, 1.5, 2 w/w% PPPO in DCM) and ternary (1 w/w% PPPO with 4, 8, 12, 16 and 20 w/w% heptane in DCM) were used (right inlet) with 1 mL pure heptane nonsolvent (left inlet). Each inlet was injected simultaneously into a confined impingement jet (CIJ) mixer at approximately 1 mL/s, to induce precipitation. The outlet mixture was further quenched into a reservoir (20 mL) of heptane, to halt further evolution of particles by kinetic arrest.

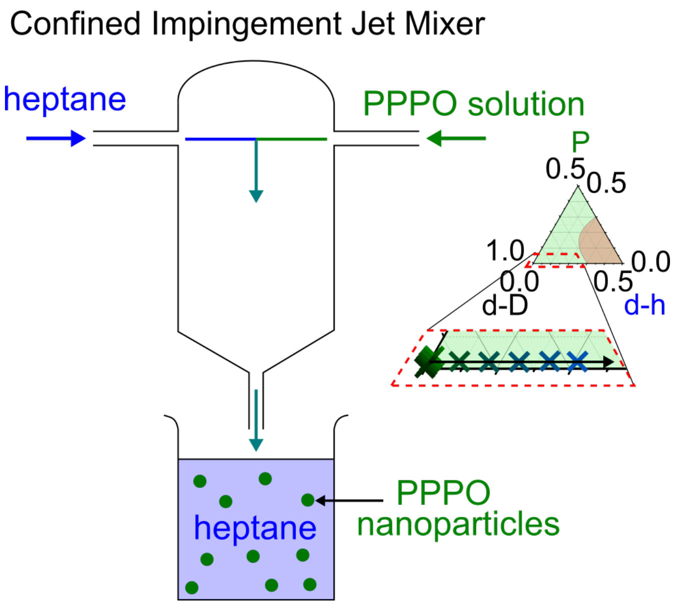

Figure S1. Schematic representation of flash nano-precipitation (FNP) with left inlet of nonsolvent (heptane) and right inlet of PPPO solution (compositions shown on enlarged section of phase diagram) undergoing high pressure mixing in a confined impingement jet mixer. The outlet stream containing precipitated nanoparticles of PPPO is further quenched into a heptane reservoir.

2 Solvent backgrounds

Figure S2 shows the SANS profiles of neat solvents d-DCM, d-heptane and a 12 w/w% mixture of d-heptane with d-DCM. These solvent backgrounds are approximately constant, with some scatter at high and low Q, and a slight downturn at high Q for d-heptane containing samples attributed to inelastic scattering effects. Polymer solution data are subtracted from the appropriate volume fraction of the actual solvent backgrounds. However, as shown in inset, solvent mixing is non-ideal and the solvent background is thus non-linear with composition.

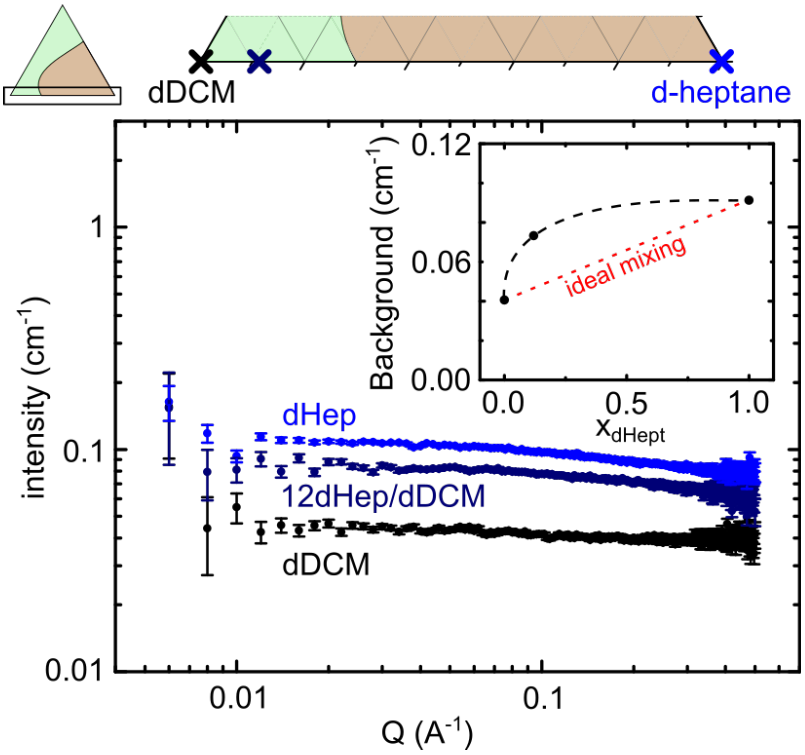

Figure S2. SANS scattering intensity of deuterated solvents, d-DCM, d-heptane and a mixture of 12 w/w% d-heptane with 88 w/w% d-DCM, compositions shown as crosses on the ternary phase diagram above, with colours corresponding to the data. Inset shows the average measured background scatter of the solvents as a function of heptane concentration (black solid circles) with a guide to the eye (dashed black line), and a comparative linear weighted average (red dashed line) expected for idea mixing.

3 Polymer background scattering

SI figure S3 depicts the linear increase in SANS background for binary PPPO:d-DCM solutions as a function of polymer concentration. The intercept corresponds to the average (coherent) scattering from d-DCM. The gradient of the linear increase yields the incoherent scattering of hydrogenated PPPO: 0.485 cm^{-1}. This value is in line with the incoherent background of numerous polymers (∼ 0.4-0.9 cm^{-1}).[3]

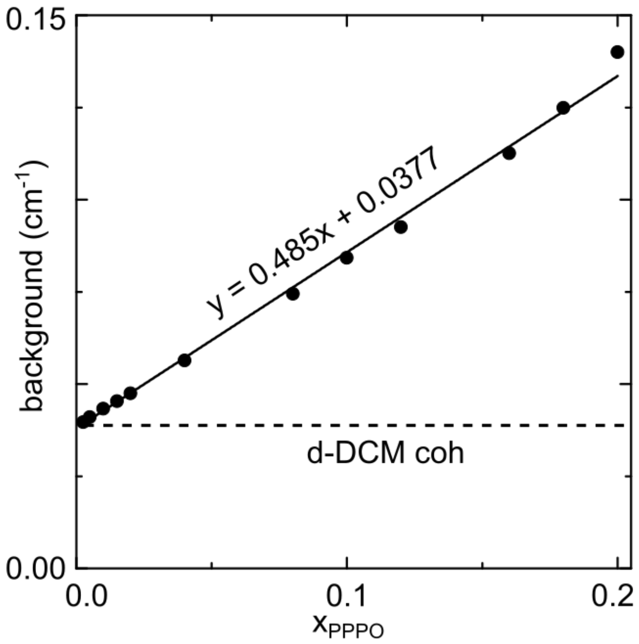

Figure S3. PPPO:d-DCM solution background obtained from data fitting, comprising a coherent contribution from the deuterated solvent (d-DCM) and the incoherent contribution from the hydrogeneous PPPO polymer. The dashed line shows the average scattering intensity of d-DCM from Fig. S2 which corresponds to the intercept of the linear fit. The slope of the fit yields the incoherent background value of PPPO, corresponding to 0.485 cm^{-1}. This value is expected for the scattering of neat PPPO (x_{PPPO}=1), in the absence of solvent.

4 Model comparison and data scaling for dilute PPPO solutions

Figure S4 (a) depicts the solvent subtracted scattering data for binary solutions of PPPO in d-DCM in the dilute regime (below c^*). Fits are shown as solid lines for both the polymer excluded volume model (red), as included in the main paper, and for the the correlation length model (black). The latter was used to estimate an effective, or equivalent, 'correlation length' for solutions below c^*, and then to compute the reduced correlation length (ξ/R_g) for the binary data set, shown in Fig. 3(a) and (b) of the main paper. Neither model, particularly for 0.25, 0.5 and 1 w/w% PPPO, fit particularly well at low Q, although the polymer excluded volume model is somewhat better. Figures S4 (b) and (c) show the data from Fig. 4(a) in the main paper without scaling, on a log-log and ln-log scale respectively. Figure S4 (b) shows that the scattering data do not precisely converge within this Q-range, due to the R_g decrease with heptane addition that effectively 'shifts' the data towards higher Q.

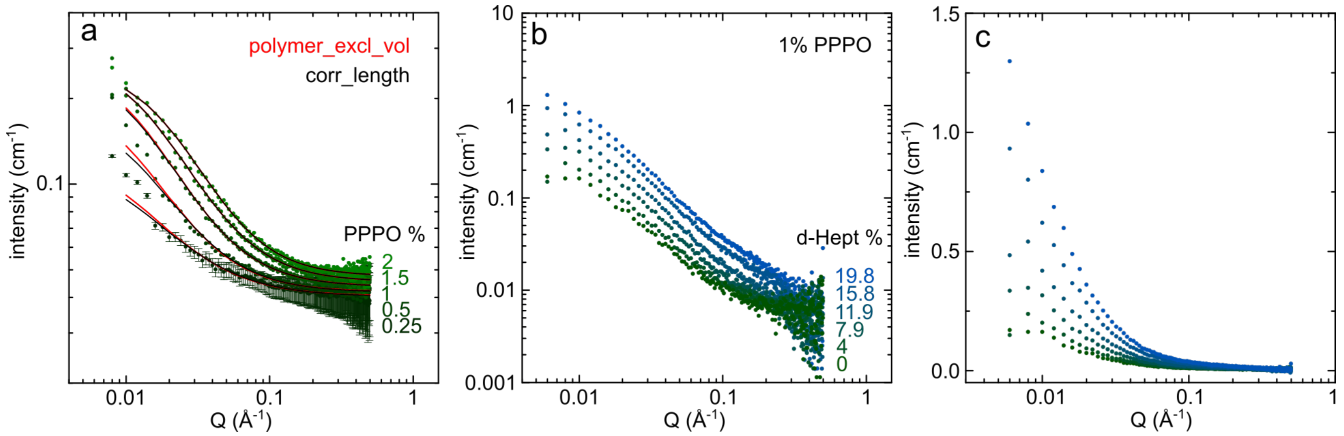

Figure S4. (a) Solvent-subtracted SANS intensity for binary mixtures of 0.25-2 w/w % PPPO in d-DCM (isopleth I below c*), with solid lines indicating model fits. Red lines show indicate the polymer excluded volume model; and black lines using the correlation length model. Error bars are included for the lowest PPPO concentration. (b) Solvent-subtracted SANS intensity for ternary mixtures of 1 w/w% PPPO:d-DCM:d-Hept (isopleth II), as a function of added d-heptane (indicated on the right), *without* the vertical scaling factor of 1.25× as shown in the main paper (log-log scale). (c) data from (b) in a lin-log scale, evidencing the increasing scattering intensity with d-heptane.

5 Time-resolved SANS near the phase boundary

Figure S5 shows that the SANS scattering intensity for the ternary solution 20 w/w% PPPO:16 w/w% d-heptane:d-DCM is not evolving with respect to time, as might have been expected during demixing or coarsening processes. These samples were, however, found to be slightly cloudy, and therefore it is possible that, at least the high M_w polymer fraction in solution, may have precipitated out. This solution was prepared approximately 2 hours before being measurement, and therefore any evolution in the solution structure will have taken place within an earlier time frame.

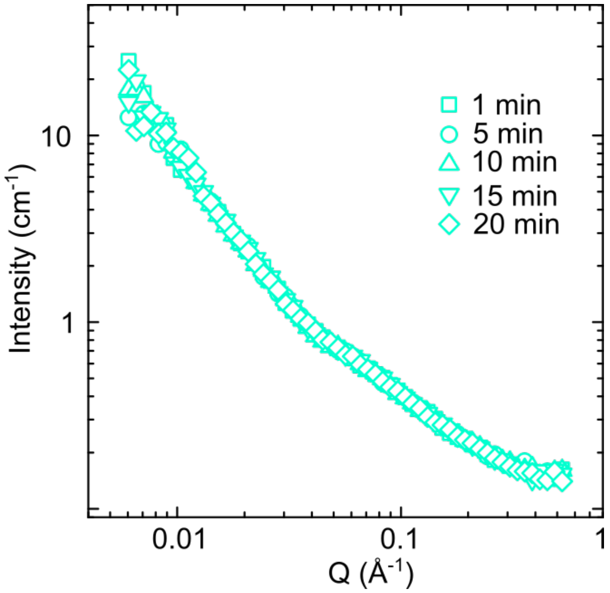

Figure S5. Time resolved SANS scattering intensity for ternary solution of 20 w/w% PPPO with 16 w/w% heptane shown at intervals of 1, 5, 10, 15 and 20 min.

6 Additional calculations

6.1 Segment length

The polymer segment length a, of a polymer in a theta solvent, is related to the radius of gyration R_g and the degree of polymerisation N by $R_{g,\theta} = a\left(\frac{N}{6}\right)^{\frac{1}{2}}$. For a good solvent, such as d-DCM in the present work, we may expect $R_{g,} = a\left(\frac{N}{6}\right)^{\frac{3}{5}}$, and equivalently in terms of molecular weight, $R_{g,} = \alpha M_w^{\frac{3}{5}}$ Where α is a constant specific to the polymer:solvent system in question, and the polydispersity of the selected polymer. For PPPO, the number average molecular weight $M_n \simeq 175$ kg/mol, weight average molecular weight $M_w \simeq 300$ kg/mol and z-average molecular weight $M_z \simeq 500$ kg/mol. The molar mass of the monomer, M_r, is 244 g/mol and the measured radius of gyration is ~ 10 nm. We thus write $N_n = \frac{M_n}{M_r} \simeq 700$, $N_w = \frac{M_w}{M_r} \simeq 1200$, and $N_z = \frac{M_z}{M_r} \simeq 2000$. The measured R_g from scattering data is a z-average R_g value. The segment length for similar polymer poly(phenylene oxide) (PPO) is found to be $\simeq 9$ Å[4]. Furthermore, the value of 9 Å is in good agreement with the calculated χ parameters when using the alternative mean-field treatment for semidilute polymer solutions[5], which computes χ_{eff} from the correlation length ξ. We thus estimate a segment length of 9 Å corresponding to roughly 3 monomers per segment, and write $R_{g,\theta} = a\left(\frac{N}{6}\right)^{\frac{1}{2}} \simeq 60$ Å.

6.2 Scattering length density

The term $\Delta\rho^2$ is the contrast factor, and is defined as the square of the scattering length density (SLD) difference between the material of interest and the medium. The SLD is calculated from

$$SLD = \frac{\sum_{i=1}^{n} b_i}{V_i}$$

where b_i is the bound coherent scattering length of ith of n atoms and V_i is the molecular volume $V_i = \frac{M_i}{\rho N_A}$, where N_A is Avogadro's constant.

atom	b (Å)
C	0.0000665
H	-0.0000374
O	0.0000580
D	0.0000667
Cl	0.0000958

Therefore, for PPPO:

$$SLD_{PPPO} = \frac{(0.0000665 \times 18) + (-0.0000374 \times 12) + 0.0000580}{355.4} = 2.27 \times 10^{-6}\text{Å}^{-2}$$

d-DCM:

$$SLD_{d-DCM} = \frac{0.0000665 + (0.0000667 \times 2) + (0.0000958 \times 2)}{107.9} = 3.37 \times 10^{-6}\text{Å}^{-2}$$

and d-heptane:

$$SLD_{d-heptane} = \frac{(0.0000665 \times 7) + (0.0000667 \times 16)}{279.2} = 5.49 \times 10^{-6}\text{Å}^{-2}$$

The contrast factor can be found as

$$\Delta\rho^2 = (SLD_{PPPO} - SLD_{d-DCM})^2 = 2.15 \times 10^{-12}\text{Å}^{-4}$$

The mixed solvent d-DCM:d-heptane scattering length densities were estimated from the volume fractions of each solvent. The relationship between SLD and heptane concentration (w/w) is shown in figure S6. The relevant compositions are shown as solid black circles.

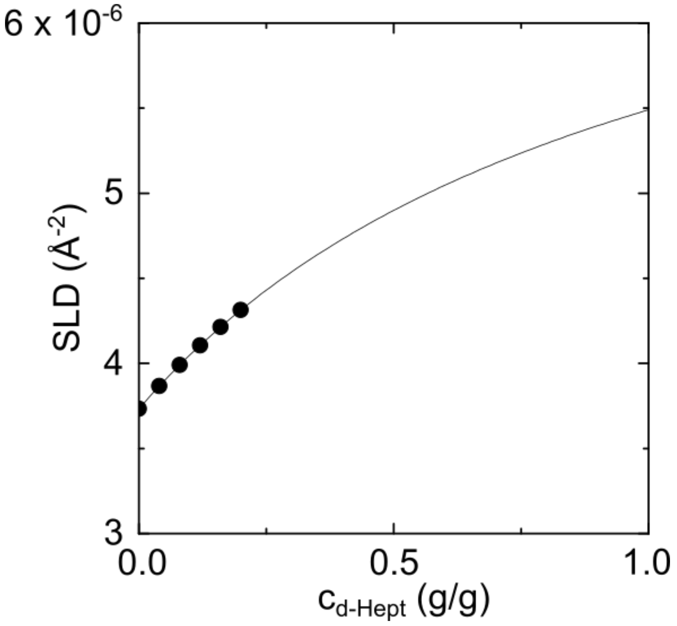

Figure S6. Scattering length density of mixed d-DCM and d-Heptane solvents (black line), calculated by volume fraction assuming ideal mixing conditions. Compositions relevant to this work are shown as solid black circles.

References

1. Saad, W. S. & Prud'Homme, R. K. Principles of nanoparticle formation by flash nanoprecipitation. *Nano Today* **11**, 212–227 (2016). DOI 10.1016/J.NANTOD.2016.04.006.
2. Sharratt, W. N., Lee, V. E., Priestley, R. D. & Cabral, J. T. Precision polymer particles by flash nanoprecipitation and microfluidic droplet extraction. *ACS Appl. Polym. Mater.* **0**, null (0). URL `https://doi.org/10.1021/acsapm.1c00546`. DOI 10.1021/acsapm.1c00546.
3. Maconnachie, A. On the assessment of incoherent neutron scattering intensities from polymer systems. *Polym.* **25**, 1068–1072 (1984). DOI 10.1016/0032-3861(84)90340-9.
4. Holland, J. R., Richards, R. W., Burgess, A. N. & Nevin, A. A Small-Angle Neutron Scattering Investigation of the Configuration of Poly(p-phenylene) Precursors in Solution. *Macromol.* **28**, 8167–8177 (1995). URL `https://pubs.acs.org/sharingguidelines`.
5. Norisuye, T. *et al.* Small angle neutron scattering studies on structural inhomogeneities in polymer gels: irradiation cross-linked gels vs chemically cross-linked gels. *Polym.* **43**, 5289–5297 (2002). DOI 10.1016/S0032-3861(02)00343-9.
